# In vivo reflectance confocal microscopy, dermoscopy, high-frequency ultrasonography, and histopathology features in a case of chromoblastomycosis

**DOI:** 10.1371/journal.pntd.0010226

**Published:** 2022-03-03

**Authors:** Gabriela A. Giraldelli, Jéssica L. C. S. Baka, Rosane Orofino-Costa, Juan Piñeiro-Maceira, Elisa Barcaui, Carlos B. Barcaui

**Affiliations:** 1 Dermatology Department, Pedro Ernesto University Hospital, University of the State of Rio de Janeiro, Rio de Janeiro, Brazil; 2 Medical Mycology Laboratory, Pedro Ernesto University Hospital, University of the State of Rio de Janeiro, Rio de Janeiro, Brazil; 3 Cliderma–Rio de Janeiro, Brazil; International Foundation for Dermatology, London, United Kingdom, UNITED KINGDOM

## Presentation of case

**Fig 1:**
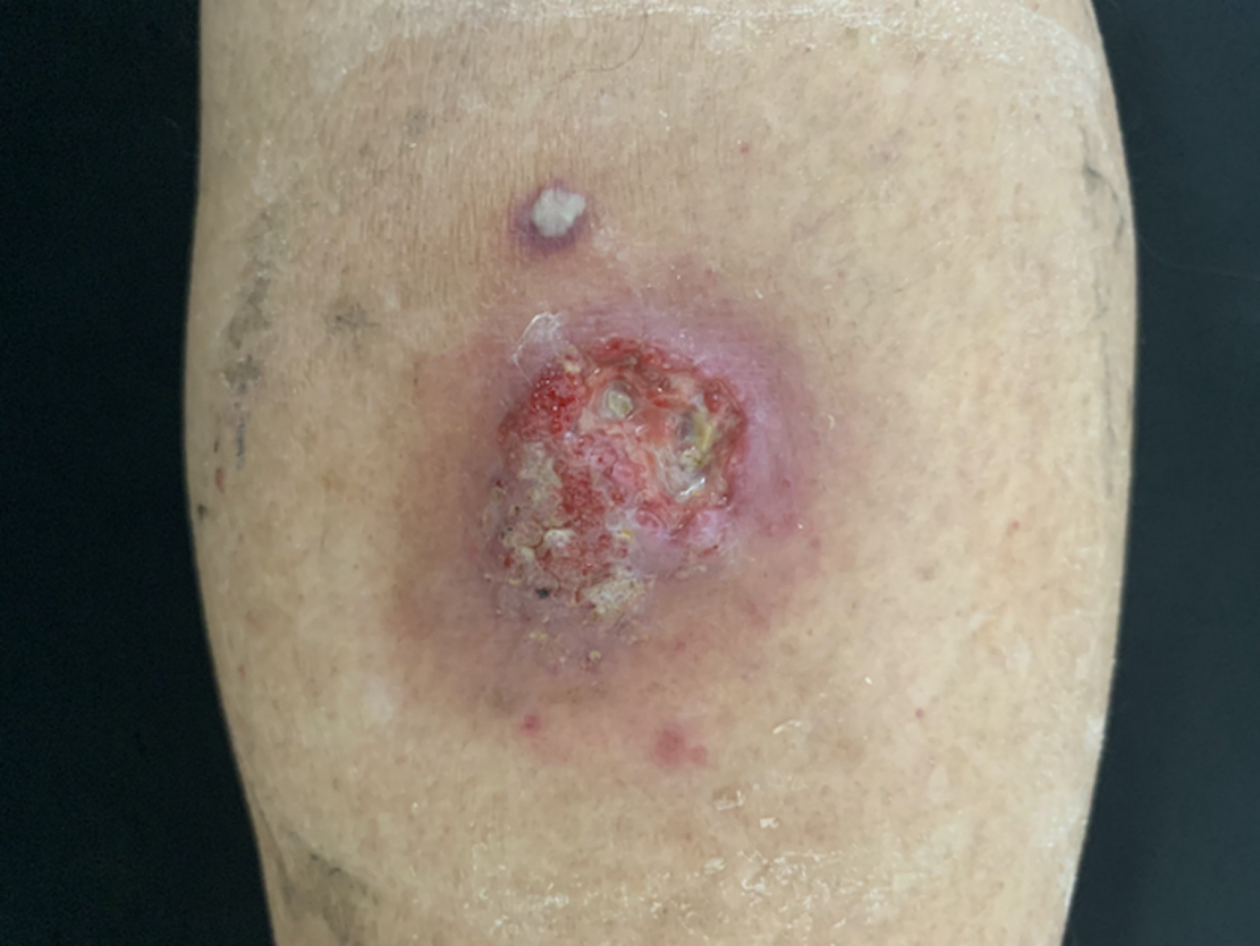
A well-defined moderate severity verrucous violaceous plaque, raised edges, measuring 4.5 × 3.0 cm with central ulceration and black dots in the periphery.

A 60-year-old male patient presented to our outpatient clinic complaining of a 12-month history of a progressively enlarging painful ulcer on his right calf. For the past 6 months, with no previous confirmed diagnosis, he had been on varying doses of systemic and topical antibiotics that resulted unsuccessfully. On examination, a well-defined moderate severity violaceous verrucous plaque with raised edges [1], measuring 4.5 × 3.0 cm with ulceration in the periphery and multiple black dots, was seen on the right calf (Fig 1).

**Fig 2:**
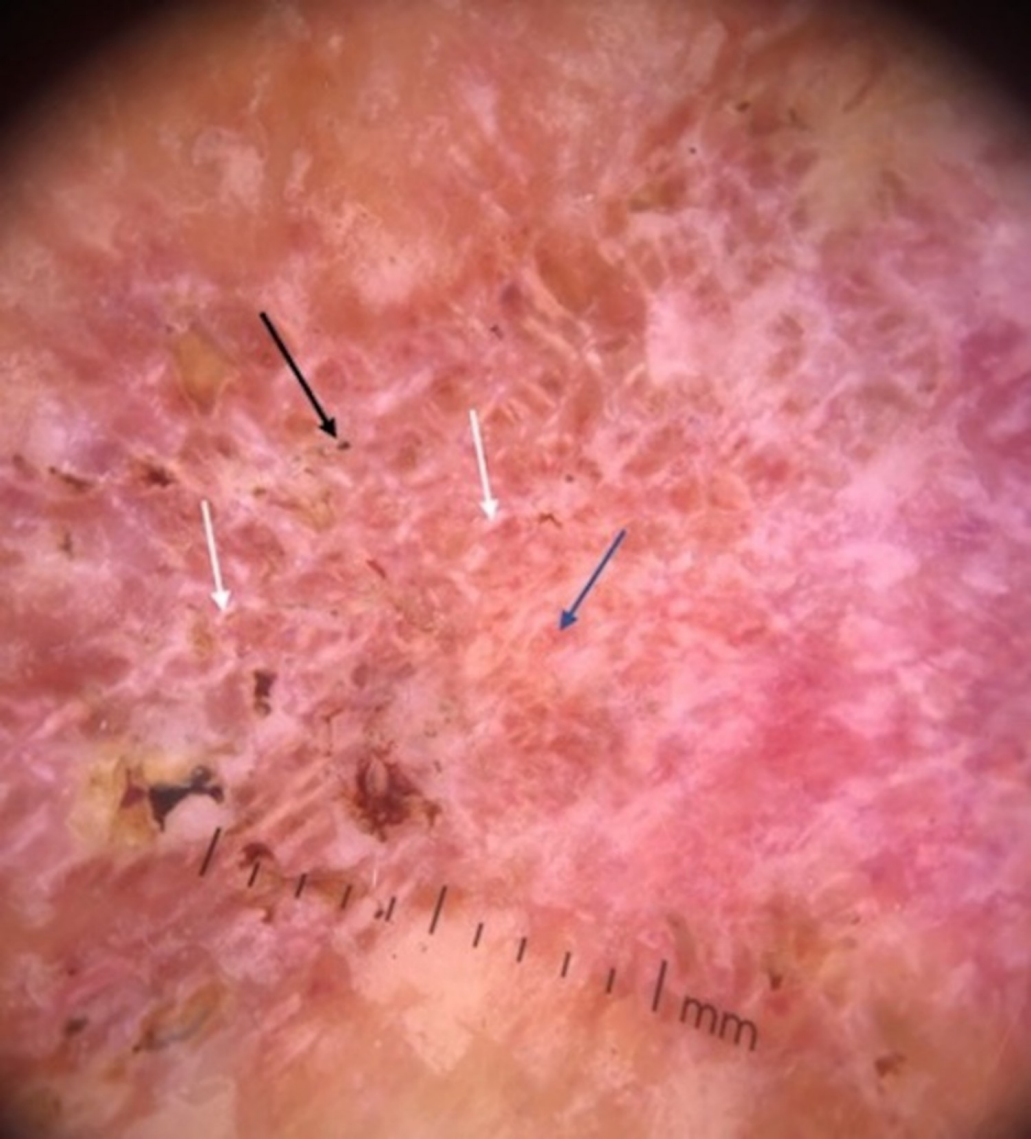
Dermoscopy shows black dots (black arrow), shiny white blotches and strands (white arrows), and yellowish-orange area (blue arrow). DermLite IV DL4; 3 Gen, polarized mode, ×10 magnification.

Dermoscopy of the inferior portion of the lesion (DermLite IV DL4; 3Gen; polarized mode, ×10 magnification) showed white scales, black dots, hemorrhagic spots, and yellow crusts at the periphery. The center of the lesion had a distinct shiny white blotches and strands and polymorphic vessels surrounding the yellowish-orange ovoid areas (Fig 2).

**Fig 3:**
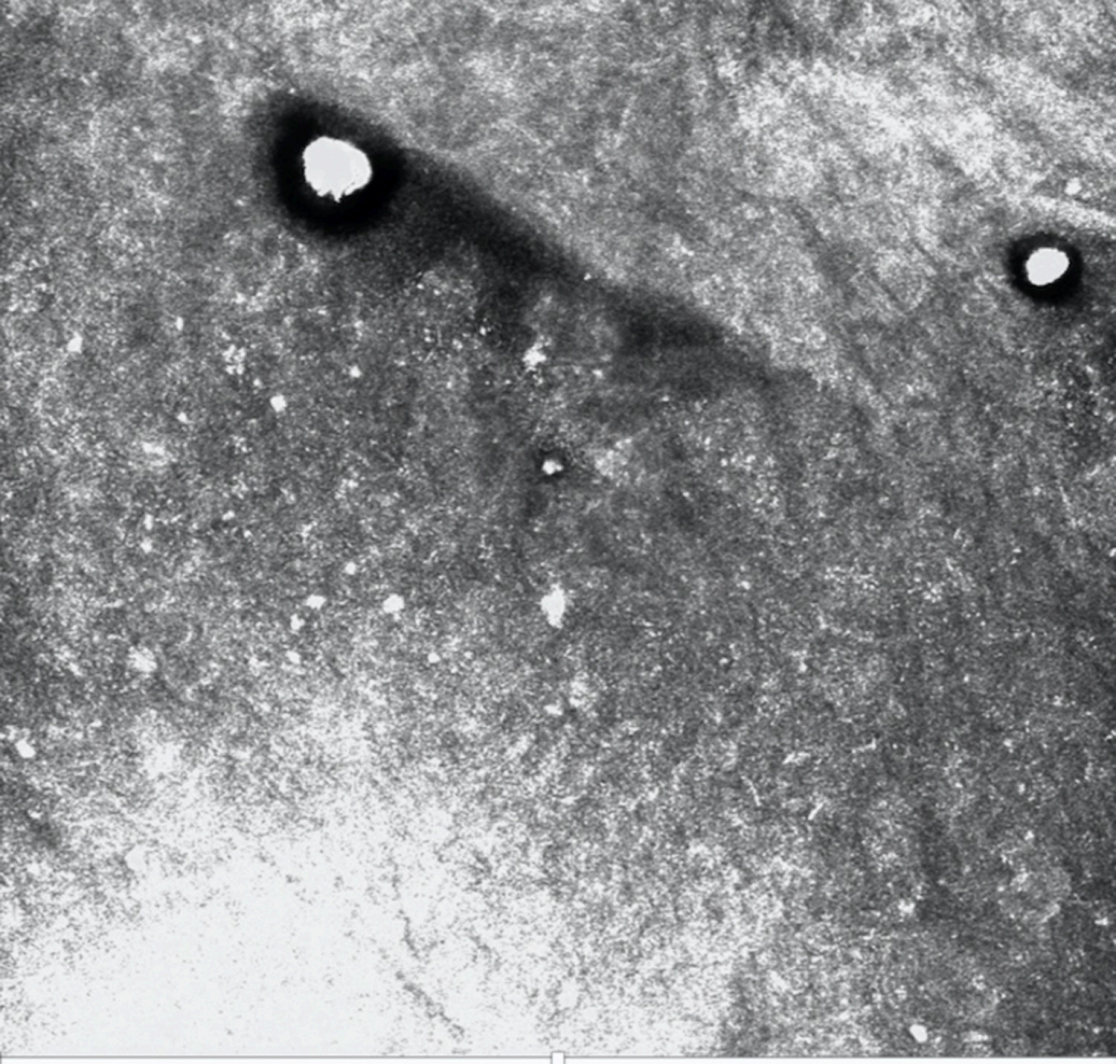
Reflectance confocal microscopy (basic image 0.5 × 0.5 mm) at the level of the stratum corneum and epidermis evidencing 2 small round hyperreflective bodies. VivaScope 1500.

On in vivo reflectance confocal microscopy examination (Vivascope 1500; Lucid, Henrietta, NY, USA), the presence of small rounded hyperreflective bodies, 0.4 μm in size, in the epidermis suggested the transepidermal elimination of fungal cells (Fig 3).

**Fig 4:**
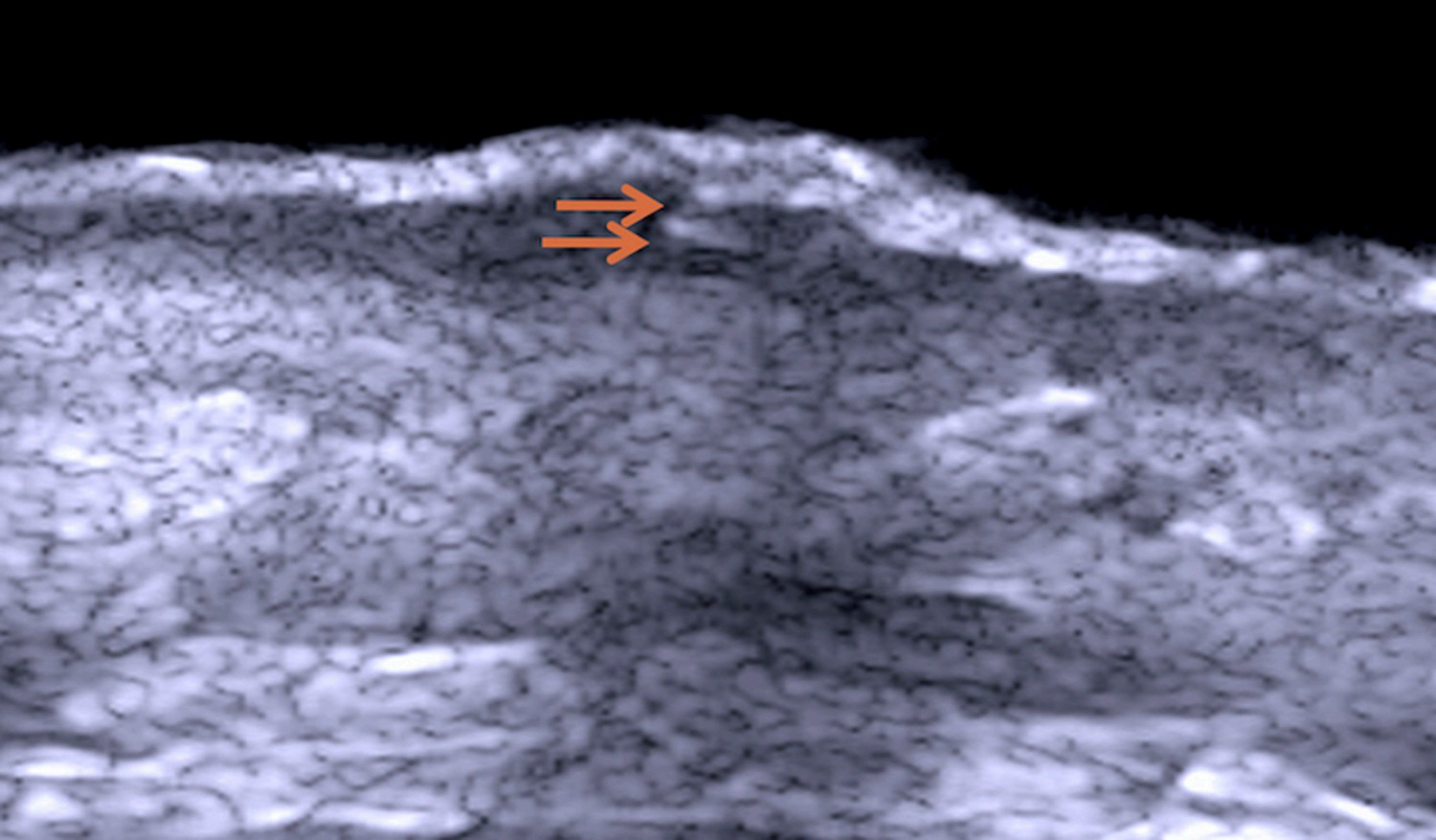
High-frequency ultrasound with Doppler shows epidermal thickening, hyperechogenic round structures (arrow), dermal fibrosis, and increased vascularization. My Lab Touch, 22 MHz linear transducer.

Ultrasonography was performed on the same area with a linear transducer using a 22-MHz frequency (My Lab Touch, Esaote, Italy). There was epidermal thickening, and 2 well-defined hyperechogenic round structures were seen in the epidermis and in the superficial dermis. Color Doppler sonography detected an irregular increase in echogenicity and vascularization giving the dermis a disorganized appearance (Fig 4).

**Fig 5:**
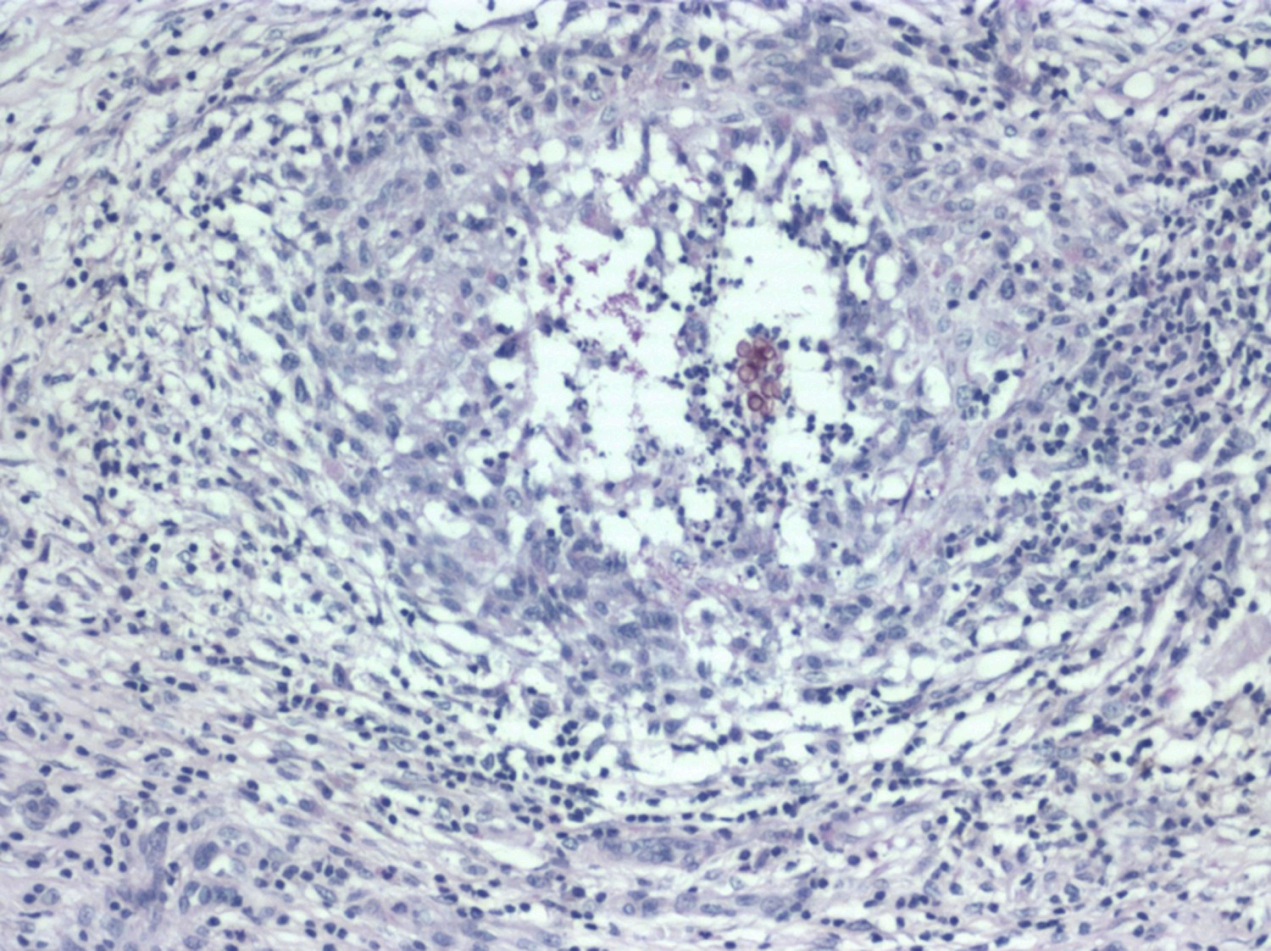
Histopathology of a skin biopsy reveals grouped fungal structures encircled by a granulomatous infiltrate in the dermis. HE, ×400.

Histopathology of a 5-mm punch biopsy from the same area showed a flattened epidermis and a granulomatous infiltrate in the dermis, with giant cells surrounding pigmented spherical fungal structures, besides an increased number of dermal capillaries and dermal fibrosis (Fig 5).

Direct mycological examination of specimens treated with KOH 20% + DMSO exhibited septate brownish muriform cells, and *Fonsecaea* sp. was isolated from tissue culture, consequently confirming the diagnosis of chromoblastomycosis.

## Ethical statement

The patient in this manuscript has given written informed consent to the publication of the case details.

## Case discussion

Chromoblastomycosis is a neglected and chronic infectious disease that has a peculiar characteristic, the transepidermal elimination of fungal structures in its early stages. Considered one of the implantation mycoses, the disease is present in several countries especially in developing countries; chromoblastomycosis is relatively common in Brazil [2]. The etiological agents are geophilic melanized fungi present in nature, particularly in rural areas. Muriform bodies or sclerotic cells, also called Medlar corpuscles, are seen as the parasitic form, and their finding is the major criterion for the diagnosis of chromoblastomycosis, either in direct mycology or in histopathology sections, differentiating this disease from phaeohyphomycosis, which is also caused by melanized fungi. Muriform bodies reproduce by cell fission, measure between 5 and 12 μm in diameter, and have a thick, pigmented wall due to the presence of melanin. The morphometric analysis of the histological distribution of infectious organisms in chromoblastomycosis revealed that elimination of 10% to 20% of all fungal elements takes place through the epidermis in skin lesions. There is a balance between proliferation and transepidermal elimination of fungal structures, which can justify the chronic evolution of the disease [3].

Dermoscopy is a helpful semiological dermatologic tool in the diagnosis of cutaneous and subcutaneous mycoses such as chromoblastomycosis. More commonly, the disease presents as verrucous slow-growing plaques with deep brown to black dots, crusts, scales, and yellow-orange characteristic structures on dermoscopy. The most important dermoscopic findings are the presence of multiple and irregular black dots, which are attributed to the elimination process of inflammatory cells, fungal elements, and hemorrhage through the epidermis [4–7]. It can be an effective and noninvasive method, assisting in the diagnosis of initial or atypical lesions [8,9]. The presence of shiny white blotches and strands is probably associated with an increased number of dermal collagen fascicles arranged horizontally and parallel to the epidermis. Collagen beams have birefringent properties that cause rapid randomization of polarized light, and that is why collagen is most notable in polarized dermoscopy [10]. In addition, through dermoscopy, we can monitor the effectiveness of the treatment, since findings such as black dots can gradually disappear during treatment, with increasing evidence of shiny white blotches and strands corresponding to the healing process [11].

There are currently several techniques of high-frequency ultrasound with many applications in the field of dermatology. Studies are underway to refine ultrasound technology and equipment, for both diagnostic and therapeutic purposes [12]. The ultrasonographic data, in this case report, led us to conclude that the dermal disorganization and the increased echogenicity are related to dermal fibrosis, and the increase in vascularization correlates with the inflammatory process observed in histopathology.

In vivo reflectance confocal microscopy is an emerging technique that cuts optically the living tissue of the skin and appendages at various depths in horizontal layers with cellular-level resolution and no change in tissue surface. This is a noninvasive imaging method; initially focused on the diagnosis of benign and malignant skin lesions, its use in basic and clinical dermatology is wide [13]. It is also suitable to follow treatment response and to the study of physiology and pathogenesis of cutaneous diseases [14]. A rising number of other indications have been recently described among which the diagnosis and management of infectious dermatological disorders [15]. In the near future, this technique will provide a fast, practical, and precise evaluation of many dermatological diseases, while studies are still necessary to create adequate guidelines and protocols for further implementation of in vivo reflectance confocal microscopy in clinical practice [16]. We propose that the 2 hyperreflective spots found in this case, measuring approximately 20 to 40 μm, indicate the high reflective power of chitin and melanin present in the fungal wall corresponding to the muriform bodies, as also seen on histopathology [17].

The authors suggest that imaging exams allow real-time, high-quality noninvasive assessment of the transepidermal elimination of fungal structures, contributing to the diagnosis and follow-up of chromoblastomycosis, notwithstanding that direct microscopy, histopathology, and the isolation of the etiological agent remains the gold standard.

**Key learning points**

➢ We propose the use of imaging as new propaedeutical tools in chromoblastomycosis.
➢ Dermoscopy correlated to clinical and histopathology findings and may be used as an aid to follow treatment response.
➢ In vivo reflectance confocal microscopy is a fast, real-time, and useful method to approach a suspected skin lesion of chromoblastomycosis.
➢ Dermoscopy, high-frequency ultrasonography, in vivo reflectance confocal microscopy, and histopathology evidenced the transepidermal elimination of fungal structures seen as black dots clinically. The contribution of these processes to a better understanding of diagnosis and therapeutic follow-up needs to be validated.
